# Angioplastie coronaire percutanée chez la femme: particularités cliniques, procédurales et pronostiques

**DOI:** 10.4314/pamj.v9i1.71223

**Published:** 2011-08-24

**Authors:** Leila Abid, Morched Hadrich, Mohamed Sahnoun, Samir Kammoun

**Affiliations:** 1 Service de cardiologie de Sfax, Hôpital Universitaire Hédi Chaker, Faculté de Médecine de Sfax, Tunisie

**Keywords:** Angioplastie coronaire percutanée, syndrome coronarien aigu, sexes, mortalité, Tunisie

## Abstract

AbstractX Les résultats de l′angioplastie transluminale (ATL) des coronaires chez la femme ont été pendant de nombreuses années controversés et longtemps considérés comme moins favorable que chez l′homme. Le but de notre travail était d’évaluer les caractéristiques de l'angioplastie coronaire chez la femme, ainsi que les résultats immédiats et à moyen terme et de les comparer à ceux chez l'homme.

Nous avons comparé 200 patients dont 100 femmes, ayant bénéficié d'une angioplastie coronaire, colligés au service de cardiologie de l'hôpital Hédi Chaker de Sfax entre 2002 et 2007.

Les femmes étaient significativement plus âgées que les hommes. La comparaison de la fréquence des facteurs de risque d'athérosclérose chez les deux sexes, a permis de noter une co-morbidité franchement plus importante chez la femme, avec une différence statiquement significative. L'ATL a été plus motivée chez la femme devant un angor stable sévère (p<0,05) et un SCA ST- (p=NS). Les femmes avaient plus d'atteinte polytronculaire (p<0,05), d'atteintes des segments moyens et distaux (p<0,05) et plus des lésions serrées (p=NS), longues et calcifiées (p<0,05). 259 stents ont été déployés, un stenting direct était plus fréquent dans la population féminine (p<0,05). Le diamètre des artères féminines, assimilé à celui du stent et/ou ballon utilisé, a été significativement moins important que celui des hommes. La longueur des stents et/ou ballon utilisés, ainsi que les pressions de larguage des stents ont été plus importantes chez la population féminine (p<0,05). Le succès angiographique global a été obtenu chez 94% de la population générale, sans différence significative entre les deux sexes. Les évènements cardiaques majeurs (MACE) hospitaliers ont été plus fréquents chez la femme (p=0,05). Après un suivi moyen de 31 mois, le taux de MACE global a été significativement plus important chez la femme (39% vs. 28%, p<0,05), portant surtout sur la mortalité globale (13% vs. 3%, p<0,05). Le taux de resténose a été comparable chez les deux sexes.

L'angioplastie coronaire constitue actuellement un moyen thérapeutique efficace et sur chez la femme, au prix de complications plus fréquentes.

## Introduction

Les résultats de l′angioplastie transluminale (ATL) des coronaires chez la femme ont été pendant de nombreuses années controversés et longtemps considérés comme moins favorable que chez l′homme. Les derniers registres montraient au contraire des taux de succès primaire et de complications identiques entre l′homme et la femme, malgré un statut clinique plus grave.

Le but de notre travail était d’évaluer les résultats immédiats et à moyen terme de l'ATL chez la femme et de comparer ces résultats à ceux de l'homme et à ceux rapportés dans les séries les plus récentes de la littérature.

## Méthodes

Il s'agit d'une étude rétrospective comparative, colligeant 200 observations tirées au hasard réparties en deux groupes: 100 femmes et 100 hommes, hospitalisés pour insuffisance coronaire et bénéficiant d'une angioplastie coronaire percutanée (ATL) au service de cardiologie de l'hôpital Hédi CHAKER de Sfax entre 2002 et 2007. Ont été inclues aussi bien les procédures à la phase aigue d'un syndrome coronarien aigu (SCA), que les procédures programmées à froid. A partir de l'analyse des dossiers médicaux nous avons rempli une fiche médicale contenant les données suivantes : paramètres épidémio-cliniques à l'admission, paramètres électro-cardiographiques à l'admission, paramètres biologiques à l'admission, protocole thérapeutique à l'admission, les modalités et les résultats de la coronarographie, les modalités et les résultats de la revascularisation myocardique, l’évolution hospitalière, l’évolution à court et à moyen terme.

### Evaluation des résultats de l'angioplastie

Le succès clinique est défini par la disparition des symptômes. Le succès angiographique est défini comme étant une sténose résiduelle post-angioplastie

**La resténose:** Est définie angiographiquement comme étant la récidive d'une sténose >50% au site dilaté. Cliniquement la resténose est définie comme une resténose angiographique symptomatique.

**La thrombose:** La thrombose aigue est définie selon ACR (195) par une occlusion aigue en intrastent (24h). La thrombose tardive est définie selon ACR (195) par une occlusion à 01 mois en intrastent. La thrombose très tardive est définie selon ACR (195) par une occlusion à 12 mois en intrastent.

Les patients ont été suivis régulièrement à la consultation externe, tout les 3 à 6 mois. A chaque consultation, un interrogatoire minutieux, un examen physique et un ECG ont été réalisées.

Une épreuve d'ischémie (épreuve d'effort ou une scinti-myocardique si EE non réalisable) a été demandé à 3, 6 mois et à tout moment devant une récidive angineuse. Un contrôle angiographique a été réalisé devant une récidive angineuse ou un test d'effort positif.

### Analyse statistique

La saisie des patients ainsi que l’étude statistique ont été réalisés grâce aux logiciels SPSS 15.0. Les variables continues exprimées en moyenne, ont été comparées par un test de Student, tandis que les variables quantitatives exprimées en pourcentage de fréquence ont été comparées à l'aide d'un test de Chi 2. Le seuil de significativité a été fixé à une valeur de p <0,05. La détermination des facteurs prédictifs de morbi-mortalité a été réalisée en analyse univariée et multivariée.

## Résultats

### Caractéristiques épidémiologiques

L’âge moyen de nos patients était de 58 ans avec des extrêmes allant de 21 à 78 ans. En comparaison aux hommes dont l’âge moyen était de 53 ans (extrêmes allant de 21 à 77 ans), les femmes étaient significativement plus âgées (âge moyen= 63,5 ans, extrêmes allant de 36 à 78); p= 0,01. D'après les données épidémiologiques (Table 1), on a noté une nette co-morbidité chez la femme : elle est plus âgée, ayant plus de diabète (64% vs 40 %; p<0,05), de dyslipidémie, d'HTA et d'obésité que chez l'homme.

**Table 1:** Caractéristiques épidémiologiques de la population

	Femmes (100)	Hommes (100)	P
Age moyen (ans)	63,5	53	< 0,05
Tabagisme (%)	11	90	< 0,05
Diabète (%)	64	40	< 0,05
Hypertension artérielle (%)	69	31	< 0,05
Dyslipidémie (%)	58	40	< 0,05
Hypercholestérolémie (%)	28	14	= 0,05
Hypertriglycéridémie	30	26	NS
Hyperuricémie (%)	8	5	NS
Coronaropathie Familiale (%)	11	11	NS
Obésité (%)	24	9	< 0,05
Antécédents d'Infarctus du Myocarde (%)	8	11	NS
Antécédents de Coronaropathie non exploré (%)	31	18	< 0,05
Antécédents de PAC (%)	4	1	NS
Antécédents d'angioplastie transluminale (%)	10	11	NS
Antécédents d'Insuffisance rénale Chronique (%)	16	3	< 0,05
Antécédents d'Accident vasculaire cérébral/Accident Ischémique Transitoire (%)	7	4	NS

### Caractéristiques cliniques

La circonstance clinique incitant la réalisation d'exploration angiographique et une angioplastie percutanée étaient motivées chez la femme essentiellement pour un angor stable et un SCA sans sus décalage segment ST. Cependant un IDM avec onde Q était la circonstance d'exploration la plus fréquemment rencontrée dans la population masculine (Table 2). L'angioplastie transluminale percutanée a été réalisée à un délai moyen de 44,77 jours chez la femme vs. 17,84 jours chez l'homme (p<0,05). Les femmes ont été dilatées plus tardivement que chez les hommes. L'Angioplastie en urgence a été prédominante chez la population masculine : 25% vs. 20%; p=0,3, tans disque celle à froid a été plus fréquente chez la population féminine : 80% vs. 75%; p=NS.

**Table 2:** Circonstance clinique motivant l'angioplastie coronaire percutanée selon le sexe

Circonstance d’ l'angioplastie transluminale	Population (N=200)	Femmes (N=100)	Hommes (N=100)	P
IDM onde Q (%)	85 (42,5%)	30 (30%)	55 (55%)	< 0,05
SCA ST (-) (%)	71 (35,5%)	37 (37%)	34 (34%)	NS
Angor stable (%)	44 (22%)	33 (33%)	11 (11%)	< 0,05

### Données biologiques

Les caractéristiques biologiques ont démontré une morbidité féminine nette. Elles ont plus d'insuffisance rénale, d'anémie, de diabète décompensé et de syndrome inflammatoire biologique. Les hommes présentaient des taux plus élevés de marqueurs biologiques cardiaques (Table 3).

**Table 3:** Répartition des données biologiques selon le sexe

Valeurs moyennes	Femmes	Hommes	P
Urée (mmol/l)	7,3	6,1	< 0,05
Glycémie>11 (mmol /l)	36%	20%	< 0,05
Clearance créatinémie (ml/mn)	69	78,8	< 0,05
IR stade I (60<Cl<90)	46%	63%	< 0,05
IR stade II (30<Cl<60)	24%	6%	< 0,05
IR stade III (15<Cl<30)	10 %	3%	< 0,05
IR stade IV (Cl<15)	2 %	1%	NS
Troponine (ng /l)	0,54	1,08	< 0,05
CPK (UI/l)	259	905	< 0,05
ASAT (UI/l)	40,45	80,9	< 0,05
Triglycérides (g/l)	1,57	1,58	NS
Cholestérol (mmol/l)	4,78	4,66	NS
CRP (mg/l)	31	10,25	< 0,05
VS	27,9	24,6	< 0,05
Fibrinogène (g/l)	2,67	2,7	NS
Hémoglobine (g/dl)	10,7	13,49	< 0,05
GB (éléments/Ul)	8390	11641	< 0,05

IR : Insuffisance rénale

### Caractéristiques angiographiques

Il n'y avait pas de différence concernant l'atteinte des artères épicardiques entre les 2 sexes. On note la dominance de l'atteinte des segments moyens (102 lésions contre 77) et distaux (42 lésions contre 26) chez la femme par rapport à l'homme avec une différence statiquement significative (p<0,05). Les femmes présentaient des lésions plus serrées, plus longues et plus calcifiées que les hommes.

L'atteinte monotronculaire était statiquement plus fréquente chez les hommes (34 (34%) contre 47 (47%), p< 0,05). Cependant les femmes avaient tendance à présenter une atteinte polytronculaire (76 (67%), 53 (51%), p< 0,05),(Table 4).

**Table 4:** Répartition des caractéristiques des lésions selon le sexe

	Population (N=200)	Femmes (N=100)	Hommes (N=100)	P
Degré de sténose des lésions (moyenne) (%)	149 (74,5%)	76%	73%	NS
Longueur des lésions (moyenne) (mm)	11,06	11,8	10 ,33	< 0,05
Présence de calcifications	23 (11,5%)	19%	4%	< 0,05
Nombre de lésion par sujet (moyenne)	2,44	2,54	2,34	NS

En conclusion, la maladie coronaire chez la femme avait tendance à être plus étendue, plus complexe, caractérisée par une atteinte des gros troncs artériels épicardiques et diagnostiquée en retard par comparaison avec l'homme.

### Caractéristiques procédurales de l'angioplastie coronaire percutanée

Au total 259 stents ont été déployés. Une angioplastie avec stenting actif a été pratiquée chez 4,5% des patients (4% femmes vs. 5% hommes, p=NS). Le nombre de stent par patient a été plus élevé chez la population féminine (1,34 vs. 1,25; p<0,05). Un diamètre de ballon et/ou stent ≤ 2,5 mm a été plus utilisé chez la population féminine : 27% vs. 10%, p<0,05. La longueur moyenne des stents et/ou des ballons utilisés est de 19,08 mm chez le groupe féminin vs. 17,07 mm chez le groupe masculin avec une différence statiquement significative (p<0,05). Les pressions de largage des stents et/ou ballon étaient plus importantes chez la femme (la pression moyenne : 17,27 vs. 16,2 atmosphères; p<0,05). L'angioplastie de segments ostiaux (29 (29%), 17 (17%), p< 0,05) ainsi que celle des segments distaux (21 (21%), 11 (11%), p< 0,05) étaient plus fréquentes chez la femme. L'ATL du segment proximal était statiquement plus significative chez les hommes (43(43%), 55(55%), p< 0,05). Il n'y avait pas de différence significative entre les deux sexes pour l'angioplastie de différentes artères épicardiques. Nous avons déduit que les lésions coronaires dilatées chez la femme sont plus longues et ayant un diamètre plus petit. La technique de dilation est plus laborieuse et plus délicate chez la population féminine (Table 5).

**Table 5:** Caractéristiques procédurales selon le sexe

		Population (N=200)	Femmes (N=100)	Hommes (N=100)	P
					
Délai moyen d'ATL (jours)		31,30	44,77	17,84	< 0,05
Angioplastie avec Stent					
	Stent Nu	185 (92,5%)	93 (93%)	92 (92%)	NS
	Stent actif	9 (4,5%)	4 (4%)	5 (5%)	NS
	Total	194 (97%)	97 (97%)	97 (97%)	NS
Stenting direct (stent seul sans prédilatation)		103 (51,5%)	60 (qw60%)	1	< 0,05
Stenting Au ballon					
	Pré-dilatation au Ballon (stent+ballon)	90 (45%)	38 (38%)	52 (52%)	< 0,05
	ATL ballon seul	15 (7,5%)	8 (8%)	7 (7%)	NS
	Total	105 (52,5%)	46 (46%)	59 (59%)	< 0,05
Revascularisation complète		86 (43%)	36 (36%)	50 (50%)	< 0,05
Revascularisation incomplète		114 (57%)	64(64%)	50 (50%)	<0,05
Nombre de stents déployés		259	134	125	NS
Nb total de stent/patient (moyen)		1,29	1,34	1,25	< 0,05
Diamètre (D) moyen de ballon et/ou stent (mm)		3	2,8	3,2	< 0,05
D ≤ 2,5 mm		40 (20%)	30 (30%)	10 (10%)	< 0,05
2,5mm<D<2,75mm		38 (19%)	20 (20%)	18 (18%)	NS
2,75 mm ≤ D<3 mm		56 (28%)	26 (29%)	30 (30%)	NS
D ≥ 3 mm		66 (33%)	24 (24%)	42 (42%)	< 0,05
Longueur moyenne stent et/ ballon (mm)		18,37	19,08	17,07	< 0,05
Pression moyenne d'inflation (atmosphères)		16,73	17,27	16,2	< 0,05

ATL : l'angioplastie transluminale

### Résultats de l'angioplastie coronaire percutanée

Résultats à la phase hospitalière :

**Succès primaire:** Dans notre série, un succès angiographique a été obtenu dans 94% de la population générale. Ce succès était observé chez 92% femmes et chez 96% hommes (p=NS). Un succès procédural (absence de MACE) a été observé dans 94,5%, avec 92 cas chez la femme VS. 97 cas chez l'homme (92% vs.97%; p=0,05). Enfin le succès clinique a été constaté chez 95 femmes et 96 hommes (p=NS), ce qui correspondant à un taux moyen de 95% dans l'ensemble de la population

**Mortalité au cours de la procédure:** Aucun décès per procédural n'a été enregistré.

**Evènements cardiaques majeurs (MACE) en intra hospitalier:** Dans notre population, il y a eu au total 5 décès durant la phase hospitalière, soit un taux de mortalité hospitalière globale de 2,5%. Parmi ces décès, 3 sont survenus chez des femmes et 2 chez l'homme, soient des taux de mortalité respectifs de 3% et de 2% (p=NS). Le sexe féminin n'a pas été un facteur prédictif de mortalité hospitalière. En analyse multivariée, l'IDM Q (0,045) et l'ACR ressuscité (0,013) ont été des facteurs prédictifs de mortalité hospitalière. 3 patients ont présenté un IDM durant la phase hospitalière (soit un taux global de 1,5%). Ces IDM ont touché 2 femmes et 1 homme (2% vs. 1%; p=NS). 3 patientes ont été redilatées ou opérées en urgence (1,5%). Le taux de MACE intra-hospitalier était plus important dans la population féminine (8 vs. 3; p=0,05). Le sexe féminin n'apparait pas comme facteur prédictif de MACE en intra-hospitalier (p=0,18). En analyse multivariée, l'atteinte polytronculaire (p=0,011) et la présence d'onde Q à l'ECG (p=0,05) étaient des facteurs prédictifs de MACE hospitalier (Table 6).

**Table 6:** Répartition taux d’évènements cardiaques majeurs (MACE) intra hospitalier selon le sexe

	Population (N=200)	Femmes (N=100)	Hommes (N=100)	P
Décès	5 (2,5%)	3 (3%)	2 (2%)	NS
IDM	3 (1,5%)	2 (2%)	1 (1%)	NS
TLR	3 (1,5%)	3 (3%)	-	NS
Total	11 (5,5%)	8 (8%)	3 (3%)	= 0,05

IDM : Infarctus du myocarde, TLR : Target Lesion Revascularisation

**Les complications per-procédurales et intra hospitalières:** Ces complications ont été notées dans 30% de la population globale. 38 % dans le groupe féminin vs. 22% dans le groupe masculin; p<0,05. D'après les données statistiques, les complications vasculaires (type hématome point ponction et/ou hématome rétropéritonéal) étaient significativement plus fréquentes chez la femme : 11 cas vs. 3 cas chez l'homme, p<0,05. Une seule patiente a présenté une hémorragie grave. Les complications rénales, étaient aussi statiquement plus importantes chez les femmes (20% vs. 4 %; p<0,05). Dans notre série le sexe féminin apparait comme facteur prédictif de complication hospitalière (p=0,021).

**Le traitement adjuvant de l'ATL et traitement de sortie :** Le traitement à base des anti GP IIb IIIa a été prescrit chez 15,5% du l'ensemble de la population, avec une prédominance non significative pour le sexe masculin (18% vs. 13%, p=NS). La durée moyenne d'hospitalisation était plus courte chez la femme : 9,24 jours vs. 10,96 jours chez l'homme, p<0,05. Il n'existait pas de différence significative entre les 2 groupes concernant le traitement de sortie.

### Résultats de suivi à court et à moyen terme

**Suivi :** Le suivi moyen était de 31,03±18,2 mois dans notre population. La durée moyenne du suivi était plus courte chez les femmes (27,85±20 mois) vs. (34,22±19,37 mois) chez l'homme, p< 0,05. L'absence de tout évènement cardiaque (angor, IDM, décès, revascularisation de la lésion cible (TLR) et revascularisation du vaisseau cible (TVR)) a été observée chez 101 patients, soit un taux de survie global sans événements de 50,1% (45% chez les femmes vs. 56% chez les hommes, p<0,05). La survenue d'un évènement cardiaque majeur au cours de suivi a été significativement plus fréquente dans la population féminine. Cette différence a été expliqué par la survenue de poussée d'IVG plus fréquente chez la femme (7% vs. 1%; p<0,05) et un taux de décès nettement plus fréquent dans la population féminine (13% vs. 3%; p<0,05). Bien que la femme ait tendance à faire plus d'IVG clinique, elle avait paradoxalement une meilleure fonction VG systolique (51,9 %±12,9 %, 51,2 %, p< 0,05). Ceci peut être expliqué par une dysfonction diastolique.

**Figure 1:**
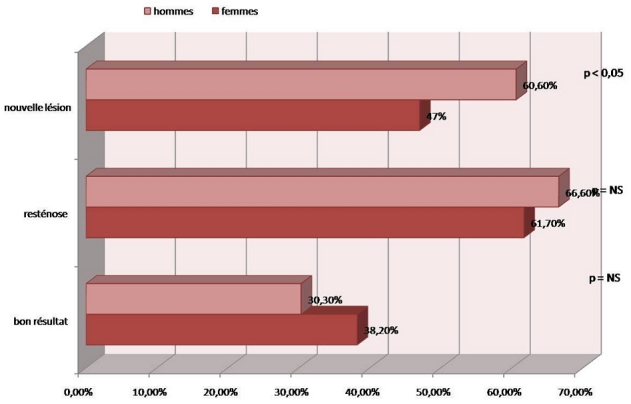
Répartition du résultat du contrôle coronarographique selon le sexe

Le contrôle angiographique n'a été réalisé au cours du suivi que chez 67 patients soit 33,5% des malades suivis, dans la majorité des cas de façon non systématique. Ils se répartissaient en 34 femmes et 33 hommes, p=NS. Le délai moyen de la réalisation de la coronarographie de contrôle était de 9,57±10 mois chez le groupe femme et de 10,21±10 mois chez le groupe homme, p=NS. Le contrôle angiographique a montré un maintien de bon résultat dans 34,3% (23/67) de la population (38,2% (13/34) des femmes vs. 30,3% (10/33) des hommes, p=NS) (Figure 1). La resténose globale était de 64% (43/67); 61,7% (21/34) chez les femmes vs. 66,6% (22/33) chez les hommes, p=NS. Le délai moyen de resténose était de 8,53±12 mois chez les femmes et de 7,8±5 mois chez les hommes, p=NS. Toutes ces resténoses étaient en intra stents nus, à l'exception d'un seul cas en intra stent actif (sexe masculin), réalisant ainsi un taux de resténose de 22% pour stent nu vs. 11% pour stent actif (p<0,05). Ce taux de resténose angiographique restait certainement sous estimé, a cause de non contrôle coronarographique systématique de nos patients.

**Figure 2:**
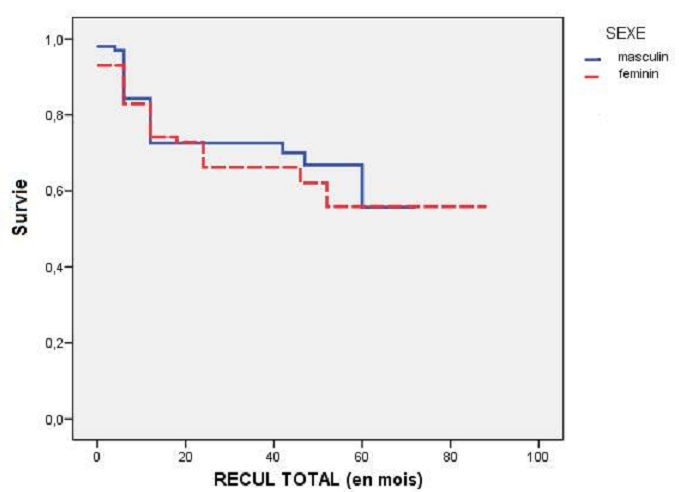
Courbe de survie sans évènements cardiaques majeurs (MACE) selon le sexe

Une thrombose aigue a été notée chez une seule patiente ayant subi une angioplastie de la bifurcation IVA/Dg et la CD par stents nus. Aucun cas de thrombose tardive, ni de thrombose très tardive n'ont été notées. Une lésion de novo a été objectivée dans 53,7% (36/67) de la population étudiée (47% (16/34) des femmes vs. 60,6% (20/33) des hommes, p<0,05) (Figure 2). Le délai moyen était de 14,38±10 mois chez la femme vs. 13,7±11 mois chez l'homme, p=NS. (Figure 1)

### Evènements cardiaques majeurs (MACE) au cours du suivi

**MACE à 1 mois :** La survenue de MACE à 30 jours a été observée chez 3 femmes et 1 hommes (p<0,05). Un seul décès chez les femmes et 2 IDM chez la femme vs 1 IDM chez l'homme. Le sexe féminin n’était pas un facteur prédictif de MACE à 1 mois (Table 6).

**MACE à 6 mois:** 29 malades ont connu la survenue de MACE à 6 mois de suivi : ils s'agissaient de 13 femmes et de 16 hommes, p=NS. Le sexe féminin n'apparait pas comme un facteur de MACE à 06 mois (p=NS). Il n'avait aucun cas d'AVC dans notre série (Table 6). En analyse multivariée, seuls l'IRC (p=0,018) et le diabète (p=0,019) ont été les facteurs prédictifs de MACE à 6 mois.

**MACE à 1 an:** Les MACEs à 1 an ont été trouvés chez 9% de toute la population (18 patients) : 9% femmes vs. 9% hommes, p=NS. Aucune différence statistiquement significative n'a été objectivée. Le sexe féminin n’était pas un facteur un élément prédictif de MACE à un 1 an (p=0,9) (Table 6). Les éléments prédictifs de MACE à 1 an, en analyse multivariée étaient : troubles de la relaxation (0,003), ATL de l'IVA (0,01), akinésie à l'ETT (0,04) et le recours aux tonicocardiaques (0,013).

**Figure 3:**
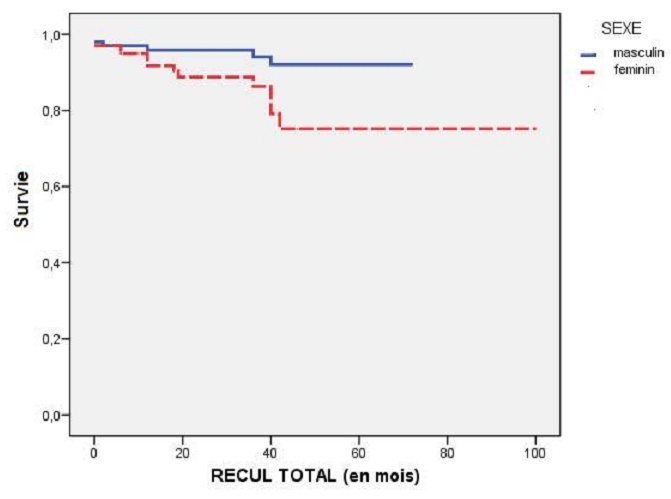
Courbe de survie (décès) selon le sexe

**MACE globaux au cours du suivi :** Les femmes avaient un taux de MACE globaux plus important que les hommes (Figure 2, Table 7). Cependant, le sexe féminin n'a pas été comme facteur prédictif de MACE global (p=0,09). En analyse multivariée, les facteurs prédicteurs de MACEs globaux étaient : l'IRC (0,004), la présence d'onde Q à l'ECG (0,006), le recours aux tonicocardiaques (0,001) et la revascularisation incomplète (0,038). En analyse multivariée, les éléments prédictifs de mortalité globale étaient : une clairance de créatinémie<60 ml/mn (0,028) et l'atteinte de TCG (0,025). Selon ces données statistiques, au cours du suivi global, les femmes avaient tendance à développer plus d’événements cardiaques majeurs. Cette différence entre les deux sexes était liée surtout au taux de décès statiquement plus élevé chez les femmes essentiellement en deuxième année de suivi (Figure 3).

**Table 7:** Répartition des d’évènements cardiaques majeurs (MACE) à 6 mois selon le sexe

	Femmes	Hommes	P
Décès	1 (1%)	1 (1%)	NS
IDM non fatal	1 (1%)	1 (1%)	NS
TLR	9/21 (42,8%)	11/22 (50%)	NS
TVR	2/21 (9,5%)	3/22 (13,6%)	NS
Total	13%	16%	NS

IDM : Infarctus du myocarde ; TLR : Target Lesion Revascularisation; TVR TLR : Target Vessel Revascularisation

## Discussion

Dans la plupart des études parues, les motifs les plus fréquents de réalisation d'une angioplastie coronaire chez la femme étaient l'angor instable et l'angor stable. Par contre, une ATL primaire était plus fréquemment pratiquée chez l'homme. En effet, l'IDM constituait un motif de réalisation de l'ATL plus fréquent dans la population masculine, avec une différence statiquement significative dans toutes les publications. Angiographiquement, dans notre série, les lésions tritronculaires étaient prédominantes chez la femme par rapport à l'homme mais sans différence significative. Les caractéristiques lésionnelles avant ATL étaient comparables entre les deux sexes, avec une longueur et un degré de sténose légèrement plus importants chez les femmes. Nos résultats s'accordent avec quelques études de la littérature [1–3]. La fréquence de l'atteinte polytronculaire chez la femme, peut être expliquée par la fréquence de la comorbidité surtout le diabète [4]. Dans le registre NHLBI, Alice et Coll. [5] ont comparé les résultats angiographiques et procédurales chez 2524 patients (1629 hommes et 895 femmes). Les données angiographiques ont montré que les femmes avaient plus des lésions calcifiées et plus d'atteinte ostiale. Cependant, les hommes avaient plus d'atteinte de bifurcation et plus d'Occlusion coronaire chronique. Concernant les caractéristiques procédurales, dans notre population, la femme avait par rapport à l'homme, une atteinte coronaire plus étendue, des lésions plus longues et un degré de sténose plus important. Ces paramètres angiographiques expliquaient le recours à des stents plus longs chez nos femmes, ce qui était en accord avec les résultats des études [1,3]. Le recours au stent actif dans notre essai (7,5%), était nettement moins fréquent que dans la littérature (91,45% dans l’étude d'Oquelli [6], et 79,35% dans l’étude de Liu Yu [3]), bien que nos femmes aient une comorbidité lourde (diabète), une maladie coronaire diffuse et des lésions longues. Ceci peut être expliqué par le faite que notre étude a été effectuée sur des patients hospitalisés entre l'année 2002 et 2007. A cette époque, la réalisation d'ATL par stent actif posait un problème économique (cout élevé), puisque la majorité de la population étudiée n'avait pas de prise en charge. Actuellement, depuis l'année 2009, ce problème s'arrange et on voit de plus en plus de patients bénéficiant de stents actifs dans notre centre hospitalier avec l’élargissement de la couverture sociale des stents actifs. Selon Alice K. Jacobs [7] et Lansky [8], les DES ont prouvé leur efficacité sur la réduction de la resténose et la revascularisation répétée chez les femmes. En plus, les avantages des stents à élutions étaient indépendants du sexe, ce qui est particulièrement encourageant [7]. Une revue de littérature [9], indique que l'apport des anti-GP IIb IIIa chez la femme reste controversé. Il est probable qu'ils soient efficaces en présence de SCA à haut risque, bénéficiant d'une attitude invasive basée sur un geste de revascularisation précoce ce qui concorde avec les recommandations de l'ESC 2007. Dans notre série le traitement à base des anti GP IIb IIIa a été prescrit chez 15,5% du l'ensemble de la population, avec une prédominance non significative pour le sexe masculin. La majorité des études parues dans la littérature, montrent que les anti-GP IIb IIIa augmentent significativement le risque d'hémorragie majeure chez la femme par rapport au placébo [10,11], excepté la méta-analyse de Cho et Coll. [12]. Ce risque accru d'hémorragie est expliqué par les auteurs de l’étude CRUSADE [13] par la tendance féminine à recevoir plus fréquemment une dose excessive d'anti GP IIb IIIa, non adaptée à la surface corporelle et à la clairance rénale (les femmes ont plus de co-morbidité et plus de tendance à l'insuffisance rénale). De même, dans une autre étude [14] le sexe féminin, le faible poids, et l'insuffisance rénale ont été identifiés comme facteurs de risque associés au surdosage par les anti-GP IIb IIIa.

Les premiers résultats du stenting dans une population de 158 femmes ont été publiés en 2000 par Fernaldo et Coll [15]. Le taux de succès immédiat était excellent pour les deux sexes (99%). Nos résultats étaient en accord avec celle de la littérature, avec des taux de succès angiographique et clinique similaires entre les deux sexes. Durant ces dernières années, plusieurs rapports ont indiqué que les femmes présentaient une incidence plus élevée des complications vasculaires après un cathétérisme cardiaque et angioplastie coronaire percutanée représentées essentiellement par les hémorragies majeures [16,17]. Dans le registre (ACC-NCDR) tout récent parue en 2009 [18], Akhtar et Coll. ont retrouvé un taux de complications hémorragiques total plus important chez les femmes. D'après Sergio Manzano et Coll. [19], dans l'analyse multivariée, l′âge de 75 ans, l'IRC, l′anémie et un traitement anti-thrombotique triple étaient des prédicteurs indépendants de saignement majeur. Les auteurs ont conclu que l'IRC était indépendamment associée à des saignements majeurs. La néphropathie au produit de contraste semble être plus fréquente chez la femme. Dans une autre étude, Lakovou et Coll [20], ont retrouvé un taux de NPC chez 23,6% des femmes contre 17,4% des hommes (p<0,0001). Ceci a été constaté malgré une proportion d'IRC avant ATL similaire entre les deux sexes (2,2% vs. 2,3%; p=NS), et une quantité moyenne de produit de contraste utilisée inférieure (267 ml vs. 287 ml; p=0,01) chez la population féminine. Les auteurs ont expliqué cette différence par la prévalence de femmes diabétiques dans leur étude (45% vs. 34%; p<0,0001). Cette idée a été confirmée par Chong E et Coll. [21] dans une étude parue en 2009. Dans notre série, l'insuffisance rénale aigue au produit de contraste post ATL, était plus retrouvée avec une différence statistiquement significative chez la population féminine.

La plupart des études qui ont comparé le taux de mortalité hospitalière après ATL chez les deux sexes, ont retrouvé un pronostic moins favorable dans la population féminine, avec une différence le plus souvent significative dans les séries anciennes [22]. Cette différence a tendance à la réduction ces dernières années [18,23].

La revue des différentes séries parues, étudiant les MACE en phase aigue post ATL, montrait leur fréquence élevée chez la population féminine [6]. Plusieurs hypothèses ont été formulées pour expliquer ce taux élevé de MACE hospitaliers chez la population féminine (fréquence du diabète [3], atteinte tritronculaire [24], anémie pré-ATL [25]).

Selon Schuhlen [26], les événements cardiaques majeurs en particulier décès et IDM étaient plus fréquent chez les femmes après 30 jours d'ATL. Ces constatations étaient en accord avec d'autres études [22,27,28]. Il apparait certes que les femmes gardent une surmortalité évidente par rapport aux hommes, mais elles tirent beaucoup plus de bénéfice de cette technique de revascularisation. Dans une publication récente « étude EASY », Helena et Coll. [23] ont rapporté un taux de MACE similaire à 1 mois entre les deux sexes. Les auteurs ont expliqué cette similitude par la prescription systémique d′abciximab en per-procédural et l'utilisation d'une thérapie antiplaquettaire maximale (l′aspirine et du clopidogrel chez tous les patients). Han-Kan Yib et Coll. [29], ont conclu que la CRP ultrasensible (hsCRP) était un prédicteur indépendant de MACE à 30 jours quelque soit le sexe. Dans notre série le taux de MACE à 30 jours a été plus élevé chez la population féminine. Le taux de CRP, bien qu'il a été statiquement plus élevé chez la population féminine (31 vs. 10,25, p<0,05), il n'a pas été déterminé chez toutes nos patientes, d'o[ugrave] on n'a pas pu conclure sur sa valeur prédictive de MACE à 1 mois.

Les résultats à moyen terme (à 6 mois) de l'angioplastie coronaire chez la population féminine sont caractérisés généralement par un pronostic moins favorable en comparaison avec ceux de la population masculine : avec une tendance à une mortalité plus importante et un taux de MACE plus élevé [30].

Les résultats à un an ne différent pas à ceux retrouvés à 6 mois, avec cependant un taux de MACE statiquement plus important chez la population féminine dans les plupart des publications [31]. Les résultats de notre série, retrouvaient une mortalité plus importante chez les femmes mais sans différence significative. Le taux de MACE était similaire chez les 2 sexes. Dans l’étude CADILLAC [31], Lansky et Coll, Le sexe féminin était un facteur prédictif indépendant de MACE ainsi que Ruchira Glaser et Coll. [32]. Cependant, dans l’étude EASY [23], Helena et Coll. n'ont pas trouvé de différence significative de taux de MACE à un an (14,1% vs 12,6%; p=NS) dans les deux groupes bien que les femmes aient plus de co-morbidités et étaient plus âgées que les hommes. Cette différence dans la survenue des MACE entre les deux sexes est expliquée par certains auteurs, par la taille réduite des artères coronaires qui contribue à des taux plus élevés de reangioplastie coronaire chez les femmes. Autre hypothèse évoquée pour expliquer ce taux plus important de MACE chez la population féminine est le phénomène de l'inflammation. En effet selon Wiviott SD et Coll. [33] certains marqueurs biologiques inflammatoires sont souvent plus élevés chez les femmes avec SCA ST (-) que chez les hommes. Ainsi, Glaser R et Coll. [34] ont conclu que les marqueurs inflammatoires étaient plus prédicateurs d′événements cardiaques majeurs chez les femmes que chez hommes au cours de suivi après ATL.

La revue de la littérature concernant la resténose après une angioplastie coronaire chez la femme est contradictoire. D'après Ellis SG [35], les femmes avaient un grand risque de resténose par rapport aux hommes. Au contraire, Mehilli et Coll. [36] ont montré un taux de resténose angiographique était également plus faible chez les femmes (28,9% vs. 33,9%, p=0,01), et ceci malgré la taille réduite des artères et la prévalence du diabète. Mehelli a expliqué cette différence statiquement significative entre les deux sexes en faveur des femmes, par l′effet protecteur des estrogènes pouvant atténuer la réponse de la paroi vasculaire causée par le ballonnet. Dans notre étude, une coronarographie de contrôle a été effectuée chez 67% de l'ensemble de la population. La resténose intrastent angiographique était constatée dans 64% (43/67 contrôlés). Il n'y avait pas de différence significative de resténose entre les 2 sexes: 61,7% (21/34) chez les femmes vs. 66,6% (22/33) chez les hommes; p=NS. Ce taux élevé de resténose au sein de notre population quelque soit le sexe pourrait être expliqué par le non contrôle coronarographique systématique sauf en cas de signes d'appel clinique, électrique et ergométrique.

## Conclusion

Quelle que soit la présentation clinique (angor stable, syndromes coronariens aigus), la population féminine coronarienne présente de vraies différences avec la population masculine. On retrouve aussi une hétérogénéité de prise en charge thérapeutique. Malgré un taux de succès comparable aux hommes, l'angioplastie coronaire chez la femme reste de moins bon pronostic. Pour améliorer cette prise en charge, la réalisation d’études dédiées nous paraît indispensable.
